# GSDMA deficiency impairs cutaneous squamous cell carcinoma growth

**DOI:** 10.3389/fonc.2026.1855268

**Published:** 2026-06-29

**Authors:** Manyun Li, Jinghan Song, Qin Gao, Qiyao Liu, Xiang Gao, Zhaoyu Lin

**Affiliations:** Ministry of Education (MOE) Key Laboratory of Model Animals for Disease Study, State Key Laboratory of Pharmaceutical Biotechnology, Jiangsu Key Laboratory of Molecular Medicine, Model Animal Research Center, National Resource Center for Mutant Mice of China, Nanjing Drum Tower Hospital, School of Medicine, Nanjing University, Nanjing, China

**Keywords:** cell proliferation, CSCC, EGFR, GSDMA, SCC

## Abstract

As a member of Gasdermin family, GSDMA is predominantly expressed in epidermal keratinocytes, hair follicle stem cells, and the upper gastrointestinal epithelium. Although it has been reported that GSDMA can be cleaved to trigger pyroptosis, its physiological function remains unclear. In this study, our results uncover a new role of GSDMA in the initiation and progression of cutaneous squamous cell carcinoma (cSCC). Deficiency of Gsdma1/2/3 strongly inhibits cSCC initiation and progression in mice. There is a significant upregulation of Gsdma1 expression in cSCC, but not Gsdma2 or Gsdma3. Mechanistically, we found that deficiency of Gsdma1/2/3 impairs EGFR-AKT axis-mediated cell proliferation during the progression of cSCC. Our findings suggest that GSDMA plays a promoting role in cSCC proliferation and may represent a potential therapeutic target.

## 1 Introduction

The gasdermin family is one of the most important protein families to trigger lytic cell death. It comprises six members in humans: GSDMA-E and PJVK. Except PJVK, the N-terminus of gasdermins could translocate onto plasma membrane and trigger pyroptosis. GSDMA is the first discovered gasdermin (1). It is mainly expressed in the epidermis and the epithelium of upper gastrointestinal tract (2, 3). In epidermis, GSDMA is highly expressed in epidermal keratinocytes and hair follicle stem cells (3).

Although GSDMA was identified more than 20 years ago, its physiological function remains unclear. Initial research on GSDMA was primarily focused on its mutant forms. Mutations in Gsdma3 induce inflammatory cell death and lead to alopecia (4, 5). The recent studies about GSDMA have consistently focused on its cleavage and pore forming function. Several reports suggest that GSDMA can be cleaved by the enzymes of pathogenic bacteria, such as group A Streptococcus and Staphylococcus aureus, and then trigger pyroptosis to initiate the host innate immune response against pathogens (6, 7). Starvation also can trigger phosphorylation of GSDMA and following pyroptosis (8). Recently, a study uncovers mitochondria−centric roles for GSDMA (9). In breast cancer, GSDMA is transcriptionally upregulated via the cGAS–STING–IRF1 axis, undergoes palmitoylation, oligomerizes on mitochondria, and promotes mitochondrial DNA release, thereby amplifying innate immune signaling.

However, GSDMA also has non-pyroptotic functions. GSDMA is mainly expressed in the upper gastrointestinal tract and the skin. The silence of GSDMA is associated with gastric cancer (3). Although GSDMA is highly expressed in epidermis, its role in skin cancer remains unknown. No previous research has reported on the role and function of GSDMA in cSCC. Recently, we found that Gsdma1 associated with epidermal hyperplasia, which is an initiating event in squamous cell carcinoma (10). Accordingly, we sought to investigate the association between GSDMA and human squamous cell carcinoma. In this study, we first revealed the relationship between GSDMA and cSCC.

## 2 Results and discussion

### 2.1 GSDMA deficiency impairs cutaneous squamous cell carcinoma growth

**Figure 1:**
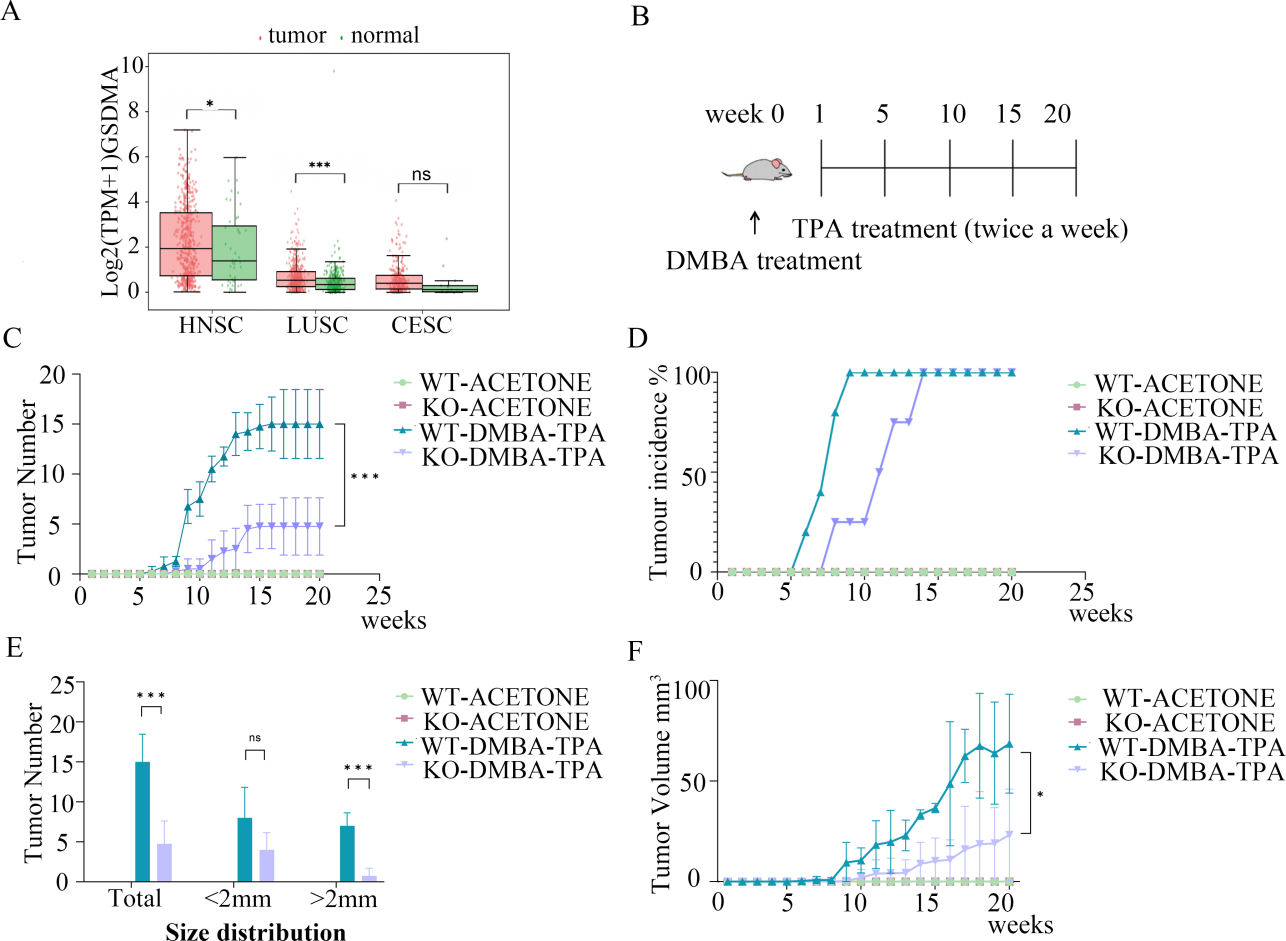
GSDMA deficiency impairs cutaneous squamous cell carcinoma growth. **(A)** Boxplot graph of GSDMA expression in carcinoma tissues and normal tissues. HNSC: head and neck squamous cell carcinoma, LUSC: lung squamous cell carcinoma, CESC: cervical squamous cell carcinoma. (HNSC-tumor: n=520, HNSC-normal: n=44, LUSC-tumor: n=498, LUSC-normal: n=338, CESC-tumor: n=306, CESC-normal: n=13). **(B)** The strategy of mouse cSCC (cutaneous squamous cell carcinoma) model. WT and Gsdma1/2/3 KO mice were treated with DMBA (dissolved in acetone) to the shaved dorsal skin. One week after DMBA application, TPA was administrated twice weekly to the same site for twenty consecutive weeks. **(C-F)** Analysis of skin tumors for wild-type C57BL/6J mice treated with DMBA–TPA (WT-DMBA–TPA), Gsdma1/2/3 KO mice treated with DMBA–TPA (KO-DMBA–TPA), wild-type C57BL/6J mice treated with acetone alone (WT-acetone), and Gsdma1/2/3 KO mice treated with acetone (KO-acetone) (n=4). **(C)** Tumor number. **(D)** Tumor incidence. **(E)** Tumor size. **(F)** Tumor volume. Results are expressed as mean ± SD. ns, not significant; *p<0.05, **p<0.01 and ***p<0.001. Statistical comparisons between two groups were conducted using Student’s t-test with *post hoc* corrections.

Although we cannot directly find the data of cutaneous squamous cell carcinoma (cSCC) by GEPIA (Gene Expression Profiling Interactive Analysis) (11), the results showed that GSDMA expression was significantly higher in head and neck squamous cell carcinoma (HNSC) and lung squamous cell carcinoma (LUSC) than in healthy tissues (**Figure 1A**). These findings suggest that GSDMA may play a role in the initiation and progression of squamous cell carcinoma.

cSCC is one of the most common non-melanoma skin cancers (NMSCs). To investigate the role of GSDMA in cSCC, we employed a well-established cSCC mouse model using DMBA and TPA (**Figure 1B**). Multiple parameters were used to evaluate the initiation and progression of squamous cell carcinoma in mice. At the early stage of tumor genesis, the latency to first observed tumor appearance was significantly prolonged in Gsdma1/2/3-deficient mice compared with wild-type (WT) controls (**Figure 1C**). From week 6, tumor incidence was markedly reduced in Gsdma1/2/3 knockout mice relative to WT mice (**Figure 1D**), indicating that Gsdma1/2/3 contributes to squamous cell carcinoma initiation or growth. Tumor number and size were further quantified at week 21 (**Figure 1E**). At this time point, both the total number of tumors and the number of tumors larger than 2 mm were significantly higher in wild-type mice than in Gsdma1/2/3-deficient mice. Furthermore, the total tumor volume of WT mice was much higher than that of Gsdma1/2/3 knockout mice (**Figure 1F**). Collectively, these data demonstrate that deletion of Gsdma1/2/3 markedly inhibits the initiation and progression of cSCC.

### 2.2 GSDMA deficiency impairs cutaneous squamous cell carcinoma growth via Egfr-Akt axis

**Figure 2:**
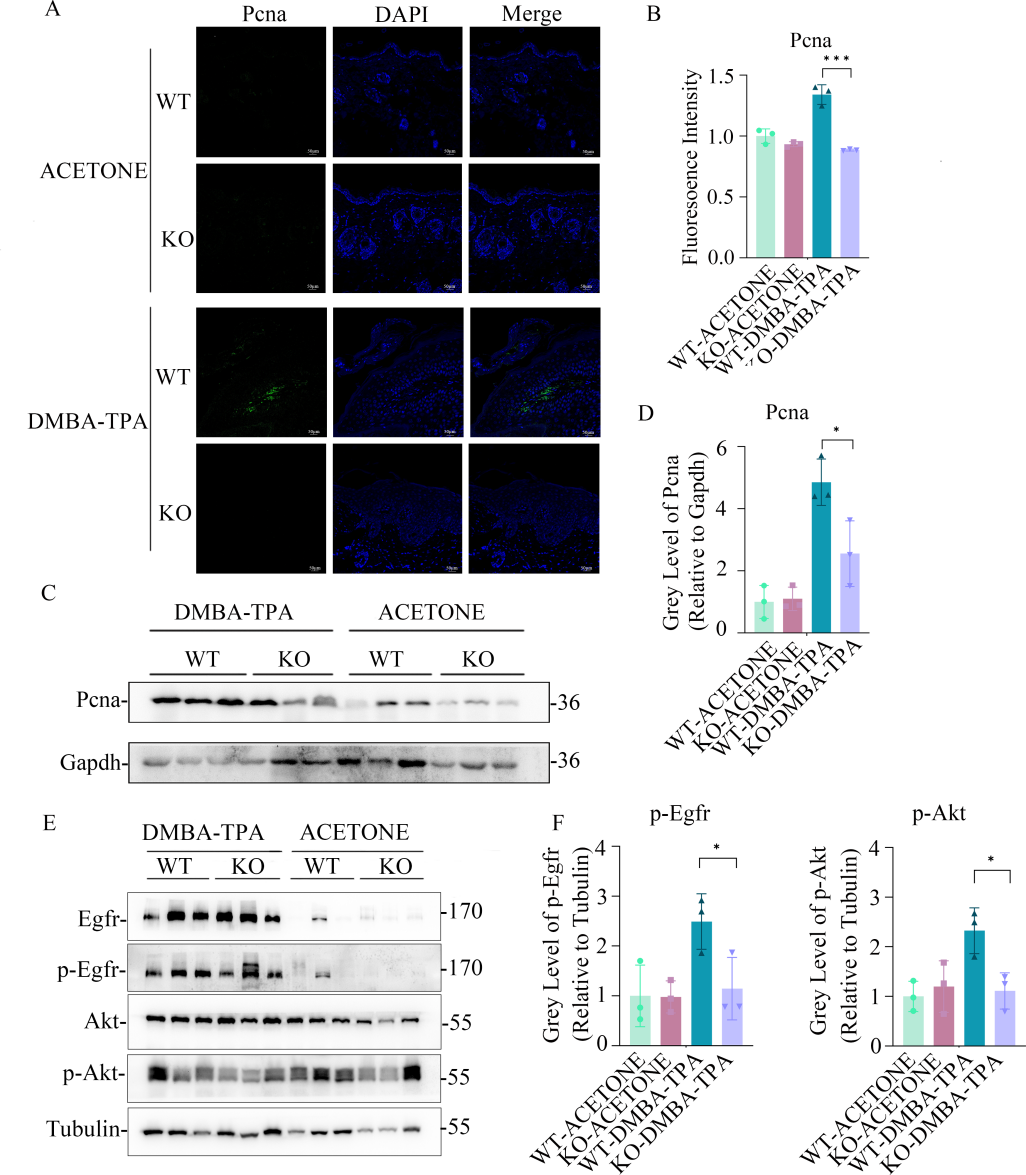
GSDMA deficiency impairs cutaneous squamous cell carcinoma growth via Egfr-Akt axis. **(A)** Immunofluorescence of Pcna in DMBA-TPA-induced cSCC and acetone-treated normal skin from WT mice and Gsdma1/2/3 KO mice. Green: Pcna, Blue: DAPI. Scale bar, 50μm(n=3). **(B)** The fluorescence intensity of Pcna in DMBA-TPA-induced cSCC and acetone-treated normal skin in panel **(A)** (n=3). **(C)** Immunoblotting of Pcna in DMBA-TPA-induced cSCC and acetone-treated normal skin from WT mice and Gsdma1/2/3 KO mice. Gapdh was used as internal control(n=3). **(D)** Grey scale analysis of Pcna/Gapdh ratio in panel **(C)**, with values normalized to the WT-ACETONE group. (n=3). **(E)** Immunoblotting of Egfr, p-Egfr, Akt, p-Akt in DMBA-TPA-induced cSCC and acetone-treated normal skin from WT mice and Gsdma1/2/3 KO mice, Tubulin was used as internal control(n=3). **(F)** Grey scale analysis of p-Egfr/Egfr and p-Akt/Akt ratio in panel **(E)**, with values normalized to the WT-ACETONE group (n=3). Results are expressed as mean ± SD. ns, not significant; *p<0.05, **p<0.01 and ***p<0.001. Statistical comparisons between two groups were conducted using Student’s t-test with *post hoc* corrections.

Previous studies have shown that upon TPA stimulation, Gsdma1/3 enhances epidermal growth factor receptor (EGFR) signaling and plays an important role in cell proliferation (10). Accordingly, we first assessed the levels of the proliferation marker Pcna in mouse skin squamous cell carcinomas (**Figures 2A, B**). We observed a significantly increased Pcna protein level in the tumor. Notably, immunofluorescence showed that Gsdma1/2/3 deficiency significantly reduced Pcna levels, indicating that Gsdma1/2/3 promotes tumor growth by enhancing cellular proliferation. To further confirm this observation, we examined Pcna protein levels in tumor tissues by immunoblotting. We found that Pcna expression was significantly elevated after DMBA-TPA treatment, but was significantly reduced in Gsdma1/2/3 KO mice (**Figures 2C, D**).

Phosphorylation of EGFR and following activation AKT play important roles in GSDMA mediated cell proliferation (10). We next investigated whether Gsdma1/2/3 accelerates cSCC progression through activation of the EGFR pathway. We analyzed the protein levels of total EGFR, phosphorylated EGFR (pEGFR), total AKT, and phosphorylated AKT (pAKT) in tumor tissues. The results showed a significant increase in pEGFR and pAKT levels in cSCC (**Figures 2E, F**). Consistent with the previous report, Gsdma1/2/3 deficiency significantly alleviated the EGFR and AKT activation, suggesting. Gsdma1/2/3 is associated with enhanced EGFR-AKT axis activation and cell proliferation.

### 2.3 Gsdma1 plays a key role in the progression of squamous cell carcinoma in mice

**Figure 3:**
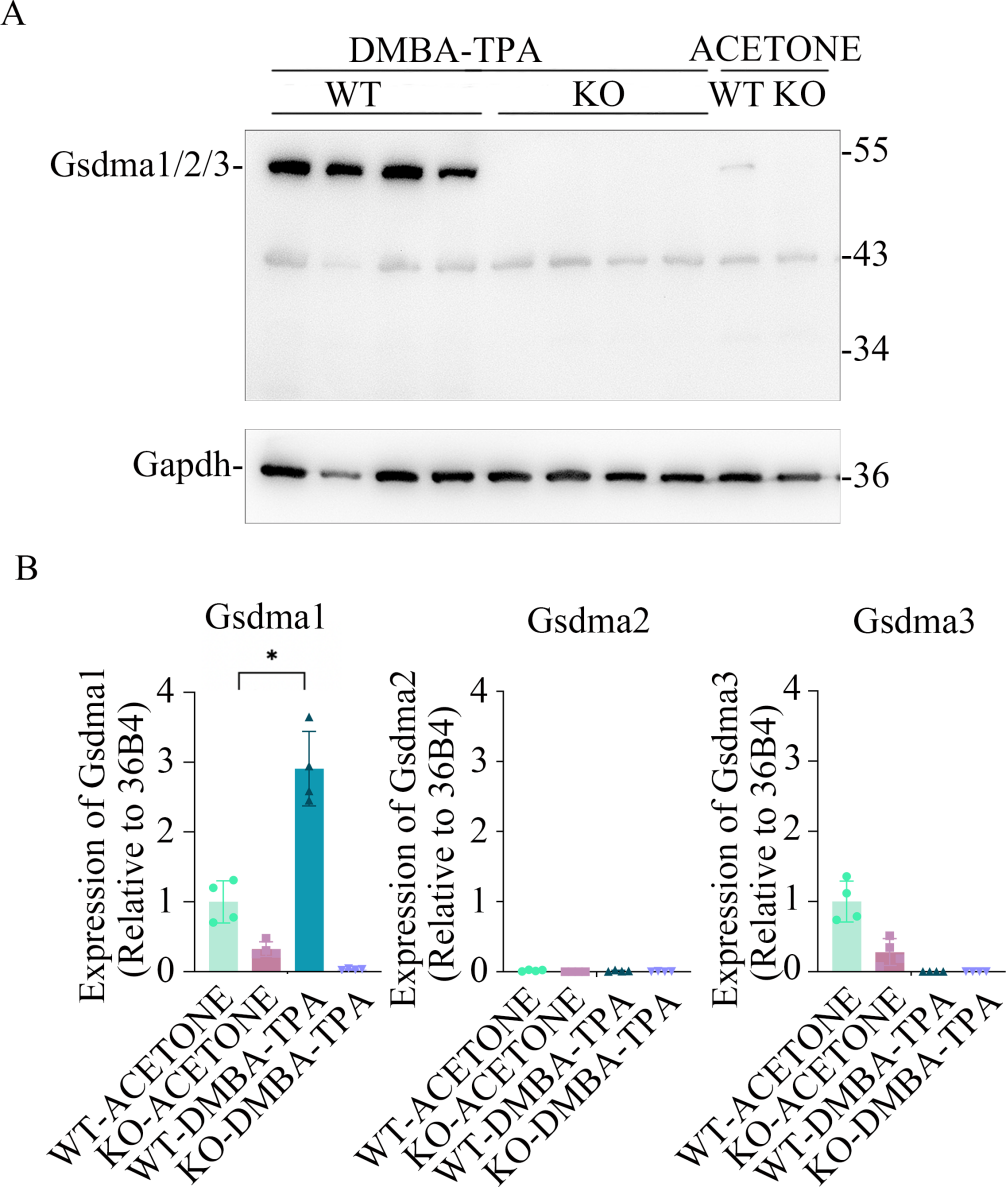
Gsdma1 plays a key role in the progression of squamous cell carcinoma in mice. **(A)** Immunoblotting of Gsdma1/2/3 in DMBA-TPA-induced tumor tissues and acetone-treated normal skin tissues from WT mice and Gsdma1/2/3 KO mice, Gapdh was used as internal control. **(B)** qRT-PCR analysis of Gsdma1/2/3 in DMBA-TPA-induced tumor tissues and acetone-treated normal skin tissues from WT mice and Gsdma1/2/3 KO mice. Rplp0 (alias 36B4) was used as internal control and the values are normalized to WT-ACETONE group. (n = 4). Results are expressed as mean ± SD. ns, not significant; *p<0.05, **p<0.01 and ***p<0.001. Statistical comparisons between two groups were conducted using Student’s t-test with *post hoc* corrections.

We next checked whether the expression pattern of Gsdma1/2/3 during the development of mouse cSCC was consistent with that found in human cancer databases. Indeed, Gsdma1/2/3 protein levels were substantially elevated in cSCC compared with control skin tissues (**Figure 3A**). Because immunoblotting cannot identify individual Gsdma homologs, we further examined their expression at the mRNA level by qPCR. The results revealed a significant upregulation of Gsdma1 expression in cSCC, but not Gsdma2 or Gsdma3 (**Figure 3B**).

## 3 Materials and methods

### 3.1 Bioinformatics analysis

GSDMA-associated tumors were analyzed in the Human Tumor database using Gene Expression Profiling Interactive Analysis (GEPIA) (http://gepia2.cancer-pku.cn/).

### 3.2 DMBA-TPA treatment

DMBA and TPA were dissolved in acetone. DMBA (25μg) was administered at a final concentration of 200μL to the shaved dorsal skin of 8-week-old mice. Then TPA (5μg) was administered in 200μL acetone to the shaved dorsal skin one week later. Mice were treated twice a week for 20 weeks. The control group was given the same dose of acetone.

### 3.3 Immunoblotting

Protein extraction was performed using RIPA buffer (1% NP-40, 50 mM Tris-HCl (pH 7.4), 0.1 mM EDTA, 1 mM DTT, 150 mM NaCl) supplemented with a protease inhibitor cocktail (P8340, Sigma-Aldrich, USA), 0.1 mM Na3VO4, 0.4mM PMSF, and NaF (0.1mM). The protein samples were mixed with 4× loading buffer and denatured by boiling at 95 °C for 5 minutes. Proteins were separated by sodium dodecyl sulfate-polyacrylamide gel electrophoresis and transferred to PVDF membranes (Amersham Biosciences, UK). The membranes were blocked with 5% non-fat dry milk (A600669-0250, Sangon Biotech, Shanghai, China) for 1 h, followed by incubation with primary antibody overnight at 4 °C. After three washes with TBST, the membranes were incubated with the corresponding secondary antibodies for 1 h. Protein bands were visualized and imaged by Tanon™ High-sig ECL Western Blotting Substrate (180-5001, Tanon, China). Band quantification was performed by ImageJ. Anti-GSDMA and anti-PCNA antibodies were purchased from Santa Cruz Biotechnology. Anti-pEGFR, anti-pAkt, and anti-Akt antibodies were purchased from Cell Signaling Technology. Anti-EGFR antibodies were purchased from Abcam Biotechnology.

### 3.4 Real-time quantitative polymerase chain reaction (qRT-PCR)

Total RNA was isolated using TRIzol reagent (R401-01, Vazyme, Nanjing, China), followed by reverse transcription into cDNA using the Hiscript III RT SuperMix for qPCR kit (R323-01, Vazyme, Nanjing, China). Real-time quantitative PCR was performed with ChamQ SYBR qPCR Master Mix (Q311-02, Vazyme, Nanjing, China) on a Roche LightCycler. And data were analyzed using the 2^(-ΔΔCT) method via Roche LightCycler software.

### 3.5 Animal studies

Eight-week-old male C57BL/6JGpt and Gsdma1/2/3 knockout mice were purchased from GemPharmatech (Nanjing, China) and housed in a specific pathogen-free (SPF) animal facility. All animal care and welfare procedures were approved by the Animal Care and Use Committee of the Model Animal Research Center, Nanjing University. Daily management checks were performed on experimental animals, and strict humane endpoints were enforced. All mice were euthanized, tumor sizes were measured and recorded, photographs were taken for documentation, and tumor and skin tissues were collected for analysis.

### 3.6 Immunofluorescence staining

At first, tissues were fixed in PFA and embedded in paraffin blocks. Then, 5-micron thick tissue sections were cut using a microtome, and applied to electrostatically charged micro slides. The sections were deparaffinized in xylenes three times for 5 minutes each. Sections were hydrated gradually through graded alcohols: wash in 100% ethanol twice for 15 minutes each, then 90% ethanol twice for 15 minutes each, finally washed in deionized H_2_O for 1 minute with stirring. Skin tissue sections were blocked for 1 h using an immunofluorescence blocking buffer prepared by dissolving 5% bovine serum albumin (BSA, 00332-0100, Sangon Biotech, Shanghai, China) in PBS containing 0.3% Triton X-100 and 0.1% sodium deoxycholate. Subsequently, PBS containing 0.1% BSA, 0.3% Triton X-100, and 0.1% sodium deoxycholate was applied to the tissue sections. A 1:200 dilution of the primary antibody was added, and the sections were incubated overnight at 4 °C. After washing, the samples were incubated at room temperature with a 1:200 diluted secondary antibody for 1h (12). Imaging was performed using a ZEISS LSM 880 confocal microscope (Carl Zeiss AG, Oberkochen, Germany).

### 3.7 Statistical analysis

The sample size (n), statistical methods, and statistical significance levels are indicated in the figure legends. All experimental data were included in the analysis; no samples or animals were excluded. Data quantification of qRT-PCR and tumor quantification were performed using GraphPad Prism 9.0. Immunoblot grayscale values were quantified using ImageJ 1.8.0. Statistical comparisons between two groups were conducted using Student’s t-test. The level of significance is indicated as p < 0.05, *p< 0.01, **p< 0.001, ***p< 0.0001, and n.s. indicates not significant.

## 4 Discussion

Our findings demonstrate that the deletion of Gsdma1/2/3 significantly inhibits the initiation and progression of cSCC. Expression of Gsdma1 is significantly upregulated in cSCC, mediating cell proliferation by enhancing the EGFR-AKT pathway, which ultimately accelerates the development of cSCC. Our findings reveal the oncogenic role of GSDMA in cSCC through EGFR-AKT-mediated cell proliferation, potentially providing a target for therapies targeting GSDMA in cSCC.

Although our results suggest that Gsdma1 is upregulated in the progress of cSCC, the signaling pathways that enhance the expression of Gsdma1 remain unknown. Additionally, our data indicate that GSDMA/Gsdma1 promotes cSCC growth through enhanced EGFR-AKT axis-mediated cell proliferation. However, the role of GSDMA/Gsdma1 in mutation efficiency and genomic stability is still unclear. Furthermore, additional mouse cSCC models are needed to confirm the function of GSDMA/Gsdma1 in the initiation and progression of cSCC. The role of GSDMA/Gsdma1 in other squamous cell carcinomas also requires further investigation.

This study relies primarily on mouse models and does not include direct validation data from human clinical samples. The main reason is the difficulty in obtaining human cSCC tissue samples. Although our study primarily focused on mouse cSCC, analyses of human tumor databases indicate that GSDMA shows abnormal expression in squamous cell carcinomas (such as head and neck squamous cell carcinoma and lung squamous cell carcinoma). Furthermore, the EGFR-AKT pathway is a core pathway in various squamous cell carcinoma (13, 14). Consequently, in human cSCC, regulation of cell proliferation mediated by GSDMA may be conserved and consistent with that observed in mice. Nevertheless, this possibility requires further experimental validation in future.

At the mechanistic level, we will further illuminate how GSDMA promotes cell proliferation via the EGFR-AKT pathway. Current findings indicate that GSDMA enhances EGFR phosphorylation and downstream AKT kinase activity. However, it remains to be confirmed whether there is a direct physical interaction between GSDMA and EGFR. And it is also unclear whether GSDMA enters the nucleus of tumor cells to regulate the expression of other genes as other gasdermins, like GSDME (15, 16). Notably, as a member of the gasdermin family, GSDMA has long been thought to be involved in pore forming and following pyroptosis. However, our study reveals that GSDMA plays a primarily proliferative role in cSCC, suggesting that its biological function is highly context-dependent. In normal epidermis, GSDMA plays a potential pro-pyroptotic role to trigger innate immunity. But during carcinogenesis, its upregulation leads to cell proliferation. Understanding the molecular basis of this context-dependent functional switch will be a key objective of future research.

Our study offers new insights for the diagnosis and treatment of cSCC. Firstly, the expression level of GSDMA may serve as a biomarker for the early diagnosis of cSCC or the assessment of disease progression, particularly in high-risk precancerous lesions (such as actinic keratosis) to predict cancerous transformation. Secondly, as a potential therapeutic target for cSCC, GSDMA can be inhibited through direct downregulation of its expression or via blocking its interaction with the EGFR signaling network to achieve anti-tumor effects. Finally, due to the dysregulation of gasdermin family members in various solid tumors (17–19), this study offers new possibilities for reconsidering the non-pyroptotic functions of gasdermin proteins in tumors.

In conclusion, this study indicates that during the initiation and progression of cSCC, GSDMA/Gsdma1 enhances aberrant epidermal cell proliferation by promoting activation of the EGFR-AKT signaling pathway, thereby accelerating tumor progression. It also suggests that GSDMA could be explored as a potential therapeutic target for cSCC.

## Data Availability

The original contributions presented in the study are included in the article/Supplementary Material. Further inquiries can be directed to the corresponding authors.
